# Editorial: Beyond cognition - adaptive technology for individualized learning

**DOI:** 10.3389/fpsyg.2024.1428974

**Published:** 2024-05-27

**Authors:** Valentin Riemer, Julian Frommel, Tina Seufert

**Affiliations:** ^1^Department Learning and Instruction, Institute of Psychology and Education, Ulm University, Ulm, Germany; ^2^Interaction/Multimedia Group, Utrecht University, Utrecht, Netherlands

**Keywords:** artificial intelligence (AI), individualized learning, adaptive learning and training systems, virtual reality, self-regulated learning (SRL), motivation

The accelerated technological progress promises to take the individuality of learning processes more and more into account. For example, the increasing prevalence of artificial intelligence (AI) applications makes it possible to adapt the learning process according to the abilities, motivation and preferencese of learners. Furthermore, immersive technologies, such as virtual reality (VR), are expanding the scope of customizable learning environments. Some frameworks for adaptive technology-assisted learning have already been proposed, including those by Shute and Zapata-Rivera (2012), Aleven et al. (2017), and Plass and Pawar (2020). These frameworks emphasize the importance of considering a range of learner characteristics and processes, including cognitive, metacognitive, motivational/affective and social factors. Nevertheless, the focus of adaptive applications has thus far been primarily on cognitive aspects such as prior knowledge (see Plass and Pawar, 2020). The aim of this Research Topic was therefore to compile current empirical work that investigates individualized technology-supported learning and focuses on learner characteristics that go beyond cognitive aspects. Specifically, the four contributions in this collection relate to learner motivation (Song and Song; Korlat et al.) on the one hand and processes of self-regulated learning (SRL) (Dever et al.; Li et al.) on the other.

In their study, Korlat et al. targeted learner motivation by integrating a game-based VR application into secondary school physics lessons. In addition to effects on isolated areas of learning performance, the VR integration showed positive effects on interest and self-concept, although these were only observed in boys. These findings demonstrate the potential of immersive technologies to enhance the appeal and accessibility of complex learning content for pupils. However, the results of Korlat et al. study also highlight the importance of aligning the technology with individual differences, in this case gender. This is consistent with previous studies on game-based learning in less immersive environments (e.g., Riemer and Schrader, 2015, 2020). One of the strengths of the study is the approach of integrating the use of the VR application into everyday school life over a longer period of time. Thereby, the study exhibits a particularly high level of ecological validity.

Similarly, Song and Song also addressed the issue of learner motivation, however, in the context of AI-supported learning with ChatGPT, focusing on Chinese students studying English as a foreign language. Besides demonstrating the positive impact of AI-supported learning on a range of writing skills, the study also highlights an increase in motivation to engage with the learning task. This corroborates previous findings on the enhancement of motivation through the interactive nature of AI-supported learning (e.g., Koć-Januchta et al., 2020). A distinctive feature of the study by Song and Song is the incorporation of qualitative data. These indicate that while learners perceive the benefits of AI-supported learning, they also recognize the challenges and the necessity to regulate the learning process.

The subject of SRL is explicitly addressed in the remaining two texts in this Research Topic. Li et al. provide a comprehensive examination of the role of scaffolding for SRL in enhancing individual learning experiences. The authors demonstrate how adaptive scaffolding, in comparison to fixed or no scaffolding, offers significant benefits to learners by promoting engagement with and mastery of learning materials through individualized support mechanisms. In particular, the results demonstrate that adaptive scaffolding leads to enhanced engagement in learning tasks, including strategic planning, monitoring, and assessment, suggesting a more self-directed approach to learning. The methodological approach utilizing the Ordered Network Analysis method provides detailed insights into the dynamics of SRL processes under different scaffolding conditions. In light of these findings, Li et al. offer valuable insights into the potential of intraindividual individualization, or micro-adaptation (see Plass and Pawar, 2020).

Finally, the study by Dever et al. is more specifically concerned with the temporal deployment of SRL operations. The authors investigate the effect of scaffolding on learners' engagement with SRL strategies in a game-based learning environment. The authors demonstrate that limiting the learner's control over their actions within the learning environment effectively supports the use of SRL strategies and improves learning outcomes. The more efficient and targeted use of SRL strategies is reflected in improved learning progress in the subject of microbiology. The methodological approach using multimodal data, including eye-tracking and log files, allows detailed observations of how learners switch between different SRL operations. In this vein, Dever et al. offer a nuanced understanding of the dynamic nature of SRL processes. Moreover, this research underscores the significance of meticulously crafted scaffolds in game-based learning environments to facilitate individual learning processes and provides guidance for educators and designers on integrating effective scaffolding techniques.

Collectively, the studies in this Research Topic address a multitude of challenges that must be overcome in field of individualized technology-supported learning. In particular, there is a necessity to transcend the limitations of traditional cognitive aspects and to incorporate an appropriate consideration of motivational, affective and social factors. This necessitates the implementation of innovative approaches, as exemplified by the works in this Research Topic. The studies by Korlat et al., Song and Song, Li et al., and Dever et al. illustrate the variety of methods and technologies that can be used to support individual learning processes. These works offer a wide range of creative solutions, including virtual reality applications, the integration of AI-supported learning and multimodal data collection. These approaches enrich the field of research by not only providing new insights into the dynamics of learning, but also offering practical implications for the design of teaching and learning environments. They demonstrate that by taking into account the individuality of learners and utilizing innovative technologies, there is the potential to improve learning experiences and enable more effective educational processes.

## Author contributions

VR: Writing – review & editing, Writing – original draft, Conceptualization. JF: Writing – review & editing, Conceptualization. TS: Writing – review & editing, Conceptualization.
